#  Rapid Point-of-Care Influenza Testing for Patients in German Emergency Rooms – A Cost-Benefit Analysis

**DOI:** 10.36469/001c.11206

**Published:** 2019-12-26

**Authors:** Roland Diel, Albert Nienhaus

**Affiliations:** 1 Institute for Epidemiology, University Medical Hospital Schleswig-Holstein, Kiel, Airway Research Center North (ARCN), Kiel 24015, Germany; 2 Lung Clinic Grosshansdorf, Germany. Airway Disease Center North (ARCN), German Center for Lung Research (DZL), Großhansdorf, 22949, Germany; 3 Institution for Statutory Accident Insurance and Prevention in the Health and Welfare Services (BGW), Hamburg 22089, Germany; 4 Institute for Health Service Research in Dermatology and Nursing, University Medical Center Hamburg-Eppendorf, Hamburg, 20246, Germany

**Keywords:** cost-benefit analysis, sensitivity analysis, PCR, POC, rapid testing, influenza

## Abstract

**Background:**

Each year, influenza causes significant morbidity and death worldwide and produces significant economic losses at the expense of the healthcare system.

**Objective:**

To assess the cost-benefit relationship of implementing a rapid point-of-care (POC) influenza test in emergency rooms (ERs) of German hospitals.

**Methods:**

A deterministic decision-analytic model simulated the incremental costs of using the Sofia^®^ Influenza A+B test compared to those of using clinical judgement alone to confirm or exclude influenza in adult ILI (influenza-like illness) patients in German ERs prior to hospitalization. Direct costs, with and without subsequent oseltamivir treatment, were evaluated from the hospital perspective as well as indirect costs incurred by nosocomial influenza transmission to hospital employees.

**Results:**

In base-case analysis, taking the influenza prevalence of 25.9% in the season 2018/2019 and assuming a hospitalization rate among influenza suspects of 21.9%, rapid testing with the Sofia^®^ followed by administering oseltamivir to patients testing positive reduced average costs of hospitalized ILI patients by €52.16 per tested patient. If oseltamivir was not offered, testing with the Sofia^®^ reduced costs by €42.28 in favor of the hospital.

In probabilistic sensitivity analysis, under all reasonable assumptions, implementing the Sofia^®^ saved on average €119.89 as compared to applying the clinical-judgement-only strategy. The major part of the cost savings, €113.17 or 94.4%, was due to the POC test’s high specificity, which led to 91% reduction in needless bed-blocking on the first day of hospitalization. However, as the sensitivity of 75.3% was only slightly higher than that of conventional clinical judgement, improved classification of patients with true influenza and a correspondingly lower rate of illness in hospital employees could not be achieved.

**Conclusions:**

Using highly specific rapid POC influenza tests in ILI patients at German ER, despite their sub-optimal sensitivity, may significantly reduce hospital expenditures.

## 1. INTRODUCTION

Seasonal influenza is a global and annually recurring public health challenge. In Germany, in the 2018/2019 season there were 182 109 laboratory-confirmed cases of influenza^1^ and the prevalence (i.e. the relative frequency of influenza culture-confirmed cases) among influenza-like illness (ILI) patients was 25.9%.^2^

Twenty two percent (21.9%) of those patients—with no difference between the proportion of ILI patients and patients with laboratory confirmed influenza—had to be hospitalized,^1^ imposing a high economic burden to the statutory public health insurances (PHI).^3^ However, as all inpatients with influenza have to be kept in costly respiratory isolation until it can be assumed that they are no longer contagious,^4^ a correct classification of the ILI as being truly caused by the influenza virus is key before making the expensive decision to isolate.

Furthermore, rapid diagnosis of influenza is highly relevant to the management of scarce economic resources. Since 1 January 2004, hospital costs in Germany are based on the German diagnosis-related groups (G-DRG) system, which assigns each influenza case to one of two categories, either to D62Z or to E79D, if the influenza is complicated by pneumonia. This imposes a fixed “base rate” of payment for 7 or 13 days of treatment. If the hospital treatment exceeds the so-called “mean length of stay”, i.e., 3.5 (category D62Z) and 6.7 (category E79D) days as calculated mathematically by the DRG Institute for Hospital Reimbursement [InEK] using case-related data of its contracted hospitals,^5^ then the G-DRG rate paid as reimbursement by the statutory health insurances usually does not cover the costs incurred by the hospital. Accordingly, in treating influenza patients covered by the statutory health insurance, hospitals should try to keep the duration of hospital stays as short as possible.^6^

In Germany, currently 81.4% of all hospitals have eliminated their in-house laboratories.^7^ To ensure the correct diagnosis, nasopharyngeal swabs or other respiratory specimen of patients suspected to have the flu must usually be sent to external labs for centralized polymerase chain reaction (PCR) testing. Those PCR influenza assays, which have near-perfect sensitivity and specificity, are often performed in batches in clinical laboratories that may be hundreds of kilometers away from the hospital introducing logistics constraints that imply a time lag of at least one day (on weekends generally two days) before the report of the test result becomes available.

Rapid influenza diagnostic tests (RIDTs)–immunoassays that detect viral antigens–have been used for diagnosis in influenza suspects in hospital emergency rooms (ERs) for many years. In comparison to the reference PCR tests, such assays are generally faster, less expensive, and easier to use, making them suitable for a non-laboratory setting. Their sensitivity, however, which usually lies below 50%,^8^ is significantly lower.

Recently, the cost-benefit of newly developed molecular or isothermal real-time influenza tests has been documented.^9^–^11^ New on the scene are improved RIDT tests providing a better sensitivity. The true cost-benefit of these improved RIDTs from the perspective of the hospital has yet to be evaluated. Our aim here is to examine whether the routine implementation of advanced RIDTs for Influenza, in an acute point-of-care (POC) setting, might lead to directly measurable economic advantages.

Given that, during the influenza season, other viruses, in particular the respiratory syncytial virus (RSV), play an important role in ILI, POC tests that are able to simultaneously detect multiple viruses are of great interest in the ER. One of the very few POC assays that enable such parallel investigations is the Sofia^®^ 2 platform (manufactured by Quidel Inc., San Diego, CA, USA).

In our model, we used the test characteristics of the Sofia^®^ Influenza A + B as performed in or within close proximity to emergency rooms (ERs) of a hospital. We compared economic outcomes of this measure to those to be expected when conventional clinical judgement alone is used to confirm or exclude influenza in patients with influenza-like illness (ILI), who would subsequently need to be hospitalized.

## 2. MATERIALS AND METHODS

### 2.1. Test System

The Sofia^®^ Influenza A+B Fluorescent Immunoassay (FIA) is a point-of-care system based on lateral flow technology that uses monoclonal antibodies labelled with Europium as a fluorescent tag. After pipetting of 120 μl of the vortexed nasopharyngeal swab out of the test tube by a fixed-volume pipette, its contents will be dispensed into the sample well of a cassette and inserted into the Sofia^®^ analyzer. The analyzer performs incubation, then measurement of the fluorescent signal, and calculates the qualitative result using assay specific algorithms. Depending on the viral load of a swab, positive results are displayed in as little as 3 minutes, negative results in 15 minutes. The final result is printed on a built-in printer or can be transferred into the laboratory information system.

### 2.2. Model Approach

Our model was parametrized by data on sensitivity and specificity of the Sofia^®^ compared to the conventional clinical approach.

After adding the contents of the pipette to the sample well of a cassette, the remaining tube containing viral transport medium could be sent to an external laboratory. For the Sofia^®^, two scenarios were considered. In the first, all ILI patients coming to the ER of a hospital during a seasonal influenza pandemic are tested with the Sofia^®^ after using a nasopharyngeal swab. Depending on the severity of symptoms, a patient would be hospitalized or discharged from the ER. In case of hospitalization, the patient would be isolated from the moment of presumptive diagnosis, given a positive Sofia^®^ test result, through the first seven days of stay (or, upon resolution of fever and respiratory symptoms for an additional 24 hours). This is to counteract the nosocomial spread of influenza, according to U.S.A^12^ and German guidelines.^13^ Given the high specificity, but only moderate sensitivity of the Sofia^®^ (95.3% and 75.3%, see below for details), follow-on molecular PCR testing of a negative patient samples is always required. As PCR testing in an external laboratory, where the patients’ samples have to be sent, ideally has both a sensitivity and a specificity of 100%, it serves to clarify whether or not the disease is due to influenza, and to correct false-negative Sofia^®^ results. In daily clinical practice, this (costly) procedure is usually not done for the mild cases in which a low viral load can be assumed and which are not considered to require hospitalization.

Under the premise that the patient arrived at the ER within the first 48 hours following the onset of his symptoms, an antiviral neuraminidase inhibitor (NI), oseltamivir, would immediately be administered to shorten symptom duration by reducing the viral load.^14^–^16^ However, as the clinical effectiveness of oseltamivir has been questioned,^17^ both options, administering or not administering oseltamivir, are calculated in our study.

In the alternative (versus Sofia^®^) scenario, i.e., the conventional clinical approach, the decision as to whether the present ILI is caused by influenza would be taken through symptom-based judgement, without rapid pre-testing. Thus, if hospitalization were required, the decision to isolate an influenza suspect and to offer NI would only be based on that clinical decision. Due to the low sensitivity and specificity of clinical judgment (see below for details), a patient sample in the form of a nasopharyngeal swab from all ILI patients judged as requiring hospitalization would be taken.

If the patient were not to be hospitalized but discharged and sent home directly from the ER, statutory health insurances (SHIs) would be charged for the costs of routine diagnostics (chest X-ray, routine laboratory values, physical examination, etc.) as well as the costs of Sofia^®^ testing, the latter according to the corresponding Physicians Fee Schedule [EBM], figure 32791.^18^ Thus, these patients whose costs are not covered by the hospital are not considered in our analysis.

In contrast, the costs for rapid testing of those patients that are ultimately hospitalized have to be paid by the hospital, and also the externally performed PCR would be directly billed to the hospital by the external laboratory, in accordance with the German Scale of Medical fees (GOÄ).

Additional costs from the hospital perspective are the so called “opportunity costs” that might occur as long as an influenza suspect is unnecessarily kept in isolation (see details below). This occurs in the cases of false-positive clinical judgement or a false-positive rapid test. Under the premise that most influenza patients are accommodated in a twin-bedded room and that hospital wards in Germany during influenza season are working at full or nearly full capacity, the economic losses caused by blocking the second bed are incurred by the hospital itself.

If a patient is isolated due to erroneous clinical judgement (no influenza present), the isolation can be ended as soon as the report of the negative laboratory PCR result is available. It is assumed that administering oseltamivir, should hospital doctors decide to do so, would be continued for 5 days^19^,^20^ if influenza infection is confirmed by the external PCR or, if the PCR result is negative, it would be stopped immediately.

It has been assumed that in most cases, patients can be discharged one day earlier than that foreseen by the DRG, given the expected response to the oseltamivir treatment.^21^ As the hospital receives a fixed DRG flat rate, this would result in an economic benefit to the hospital.

If the Sofia^®^ is used as described here, a false-positive test result would not be corrected (like the clinical judgment would) after 1–2 days, because no external PCR test would be performed. Under the worst-case assumptions, patients that falsely tested positive would end up being isolated for 7 days and ineffectively receive NI for 5 days. Early release (one day earlier) is, therefore, out of the question.

Our model also considers the economic effects of patient-to-caregiver transmission that occurs when undetected influenza patients infect susceptible healthcare workers (HCW) (those waving vaccination or having ineffective vaccine protection). We incorporated a secondary attack rate, with the measured effect being sick days for hospital workers, the costs of which, under the German system, is borne by the hospital.

### 2.3. Model Structure

**Figure 1:**
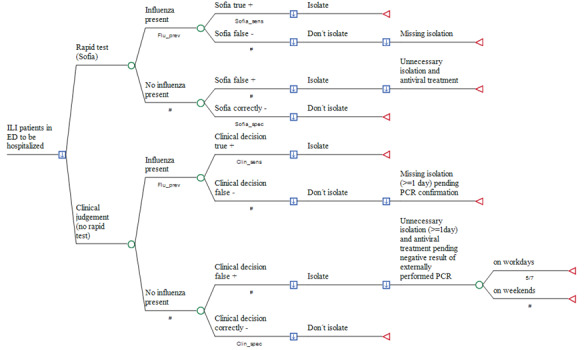
Rapid POC testing versus the conventional approach in influenza suspects prior to hospitalization. Legend to figure 1: A decision node (square) indicates a choice facing the decision maker or the consequences of a decision. Branches from a chance node (circles) represent the possible outcomes of an event; terminal nodes (triangles) denote the endpoints of a scenario and are assigned the costs of a prior series of actions and events. The arrows in the decision notes pointing downwards demonstrate that the optimal path of the model is that with the lowest total cost. #: Complementary probability (all probabilities of chance node’s branches to sum to 1.0); +: positive; −: negative. ED: Emergency department

Two deterministic, patient-based, decision-analytic models were developed, simulating the incremental costs of using Sofia^®^ in adult patients who attend the ER of a German hospital with acute moderate-to-severe respiratory infection and suspicion of influenza, compared to conventional clinical judgement. As rapid testing in those patients who will be discharged and sent home from ER paid by the local Association of Statutory Health Insurance Physicians (Kassenärztliche Vereinigung, KV) and external PCR is not required in such mild cases, the decision tree is restricted to patients due for hospitalization. Accordingly, the perspective taken is that of the hospital only (see Figure 1).

The treatments compared were as described above: (1) empiric clinical investigation without testing for viruses, or (2) real-time influenza testing used to guide antiviral treatment and the decision as to whether a patient (if hospitalization is required due to signs of severe lower reparatory infection) requires isolation. In the first model, total costs of outcomes were simulated for each study arm, including (1) the medical cost of Sofia^®^ testing and administering oseltamivir; (2) medical costs of external PCR testing in all clinically judged patients without rapid testing prior to hospitalization; (3) opportunity costs due to blocking a twin-bed reimbursement per day of hospital stay within the fixed payment DRG period; (4) reimbursement per day of hospital stay within the fixed payment DRG period; and (5) sick pay costs at the expense of the hospital if secondarily infected by hospitalized but unrecognized influenza patients. In the second model, oseltamivir was not administered.

We used the TreeAge Software (TreeAge Inc. Williamstown MA, USA) for model building and analysis and examined our inputs over a wide range in sensitivity analyses to identify influential factors that would alter the base-case findings. First, univariate sensitivity analysis was performed using all variables to examine the extent to which our calculations were affected by the varying selected assumptions. Variation was done using either (a) the lower and upper bounds of a parameter’s standard deviation or (b) those of its 95% confidence interval. Where these are not applicable, our model simply causes parameter values to vary by ± 20% of the base-case value, according to international practice, unless stated otherwise.

Furthermore, and in order to capture the interactions between multiple inputs, we provided a probabilistic sensitivity analysis (PSA) by assigning an appropriate statistical (probability) distribution for all parameters, randomly drawn in a second-order Monte-Carlo simulation (n = 10 000). All costs are reported in 2019 Euros (€).

Input parameters are shown together with their probabilistic distributions.

### 2.4. Model Input

#### 2.4.1. Epidemiological and Laboratory Parameters

##### 2.4.1.1 Prevalence of Influence in Season 2018/2019

According to the data provided by the RespVir-net,^2^ there were approximately 49 604 findings of patients presenting flu-like symptoms during this period, of which 17 878 were virus-positive and 31 726 virus-negative. Of the 17 878 virus-positive findings, approximately 12 858 samples, or 71.9% of the virus-positive samples, were identified as influenza (Flu A and Flu B taken together). In relation to all tests conducted on symptomatic patients (virus-positive + virus-negative), the rate of Flu-positive results was 12 858/49 604, or 25.92% (95% CI, 25.5 to 26.3%).

##### 2.4.1.2 Sensitivity and specificity of the Sofia^®^

According to the results of a recently published meta-analysis,^8^ the combined sensitivity and specificity of the Sofia^®^ for Influenza A and B is 75.3% [95% CI, 59.2 to 91.5%] and 95.3% [95% CI, 91.5 to 99.2%].

##### 2.4.1.3 Sensitivity and Specificity of clinical judgement

In Yang’s South-Korean study^22^ involving 1417 patients during the influenza season 2011/2012, clinical judgement showed a sensitivity of 71.3% (95% CI, 65.4 to 76.7%) and a specificity of 60.1% (CI 95%, 57.2 to 63%) versus laboratory confirmed influenza. We used the figures of Yang et al. as the baseline.

##### 2.4.1.4 Rate of Hospitalizations

According to the most recent data of the German Robert Koch Institute,^1^ a total of 182 109 laboratory-confirmed influenza infections were reported for the 2018/2019 season. Of those influenza cases, the rate of patients requiring hospitalization was 39 945/182 109, or 21.9.0% (95% CI, 21.7%–22.1%).^1^ This was slightly lower than the estimate of 23% for the preceding season 2016/2017.^28^ The lowest estimate generated for one of the last five years was for the 2014/2015 season, at 15.7% (11 000/70 000).^29^

Of note, as explicitly mentioned in the Robert Koch Institute (RKI)-survey^1^ and stated in the literature,^30^ the necessity of hospitalization depends on how severe the disease is in a particular patient. Thus, it can be considered the same for influenza and every other acute respiratory infection, during the influenza season. Accordingly, we used the value of 21.9% hospitalized patients as the base-case estimate for all influenza suspects and took the value of 15.7% as the lower bound and for the value of 23% as the upper bound in the sensitivity analysis.

##### 2.4.1.5 Duration of stay in hospital by antiviral use

According to Aoki’s model et al.,^31^ early administration of oral oseltamivir corresponded to a benefit of ~10 h (range 8–15) shorter duration of illness for every 6 hours earlier that the treatment was initiated. Thus, it seemed plausible that, as noted above, the duration of disease and thus the absolute number of days spent in a hospital ward before being discharged can be reduced by at least one day when drug administration is started immediately.

##### 2.4.1.6 Vaccination rate and its effectiveness among HCW

Despite its known benefits, compliance with vaccination recommendations is low among health care personnel in Germany. According to a 2017 survey by the Robert Koch Institute, only 40.1% of all HCW have been vaccinated against influenza.^27^ The highest rate was that among physicians (61.4%), the lowest at 32.5%, among nurses. However, despite all efforts towards increasing vaccination coverage, it should be taken into consideration that vaccine effectiveness may vary, depending on how well the prevalent circulating viruses are matched to the influenza vaccine in the respective season. The 2018 Cochrane review, which evaluated randomized controlled trials (RCTs) or quasi-RCTs comparing influenza vaccines with placebo versus no intervention, found that influenza vaccination in healthy adults aged 16 to 65 years has only a modest effect in reducing the number of influenza cases.^32^

Indeed, during the 2017/2018 season, as in the 2015/2016 season, vaccine effectiveness in Germany, adjusted to age, gender, week of onset of the disease, and pre-existing illness, was only 15% (95% CI, 15 to 37%).^1^ Assuming a vaccination rate of 40.1% as the base-case value for our model, thus, leaves about 94% (1−(0.401 × 0.15)) of hospital personnel susceptible to influenza infection. Our best-case assumption for sensitivity analysis was based on the effectiveness value of 49% (95% CI, 17 to 49%) for the influenza season 2011/2012,^33^ resulting in a proportion of about 80.4% (1− (0.401 × 0.49)) unprotected hospital employees.

##### 2.4.1.7 Secondary Attack Rate after Influenza Virus Transmission

Healthcare personnel may encounter considerable concentrations of influenza virus when in close proximity to patients. According to the study of Savage et al., the secondary attack rate (SAR)–defined as near contact with new onset of acute respiratory illness–was estimated at 20.2% (95% CI, 15.4 to 25.6%), falling mostly within 3 to 7 days following symptom onset of the index case.^23^ As studies explicitly providing SAR rates among HCW infected by patients are lacking, we used this figure derived from household contacts as proxy for transmission in hospital.

#### 2.4.2 Economic Parameters

According to the information provided by Quidel Inc. (personal communication) the costs of the Sofia^®^ are uniformly €12 (without any volume discount) and include the provision of the required instrument at no charge by the manufacturer.

The figures for the other economic parameters are listed in Table 1; their origins have been published in detail elsewhere.^9^

Briefly, the cost paid for having an influenza-PCR test performed in Germany comes to €44.88. The opportunity cost of blocking a twin bed is €350.19 per day. While the cost of oseltamivir administered for a period of 5 days is €12.37, the average reimbursement per bed for an influenza patient (G-DRG D62Z based on ICD-10 Code J10.1) that may be saved by early discharge (one day) of an influenza patient thanks to the clinical effect of the oseltamivir treatment amounts to €233.46. We assume that when an unprotected HCW, infected by an unrecognized influenza patient, will on average miss 7.2 days of work, multiplied with a productivity loss of €156.99 per day.

## RESULTS

In the base-case analysis, utilizing the Sofia^®^ test in ILI patients with subsequent oseltamivir treatment is on average €52.16 less costly per eventually hospitalized patient, compared to the conventional clinical approach (see Table 2), even though all negative results have to be re-checked by an external PCR.

Univariate sensitivity analysis, in which all variables in the decision are changed between plausible extremes ranges, reveals that, above all, the cost saving is attributable to the principal advantage of the Sofia^®^ that hospitals can dramatically reduce the number of patients to be placed in isolation upon admission as in-patients (while also reducing, if only marginally, the incidence of in-hospital transmission of influenza).

The technical characteristics (i.e. specificity and sensitivity) of a diagnostic test are determinant of its economic value. The threshold of 95% specificity is often crucial for a POC test, as even a small decrease in specificity of the Sofia^®^ (by 2.9% from the base case value of 95.3% to 92.4%) would result in a reversion of the relative cost savings occurring by utilizing the Sofia^®^ as an add-on diagnostic test (Table 3). Variations of all other parameters do not change the result of the base case analysis and only increase or decrease the absolute amount of lower expenditures in favor of the hospital.

As can be seen in Table 3, an increase in sensitivity of the Sofia^®^ by 16.2% from the baseline of 75. 4% up to 91.5%, the upper bound of the 95% CI, would result in additional relative savings of €17.52 (€52.16 minus €34.65) in favor of the hospital. In contrast, only a small increase of 4.8% in specificity of the clinical approach from the base case value of 57.2% to 63.0% would reduce the economic advantages of rapid testing by €9.57 (€52.16 minus €42.59).

The variation of the opportunity costs per day (blocked second bed in a twin-bed room) within the range of ± 20% has only a marginal impact on the cost savings. For example, an increase in opportunity costs to €420.23 (our upper bound) would only result in additional savings of €19.10 (€62.16 minus €43.06) when using the rapid test as opposed to the conventional clinical approach in our model.

The economic impact of all other variables appears to be negligible. Of note, the cost reductions by implementing rapid testing are not dependent on the actual prevalence of influenza in the investigated population of ILI patients. Varying between a prevalence of 20% and 46.2% (i.e. a difference of more than 26%) reduces the cost savings achieved by utilizing the Sofia^®^ only by the very small amount of €4.69.

It should also be pointed out that when using the Sofia^®^, early oseltamivir treatment following a positive test result only plays a minor role. Due to the considerable proportion of 24.7% influenza patients tested false-negative by the Sofia^®^, the savings in favor of the hospital amount to only €4.33 per patient compared to a scenario where no neuraminidase inhibitor (NI) will be offered. In those patients tested as false negative, NI treatment will not be initiated immediately (i.e. before the falsely negative test result is corrected by the later external PCR testing). Also, the marginally higher sensitivity (75.3% versus 71.3%) of the Sofia^®^ compared to the conventional clinical approach does not make a significant difference in the number of patients scored correctly positive for whom oseltamivir could, in principal, be offered.

In probabilistic sensitivity analysis (PSA), performing rapid POC testing on each patient prior to hospitalization reduces the costs occurring under the conventional clinical approach, even at the expense of the hospital, by €119.89 (see Table 4). Of note, testing with Sofia^®^ is consistently less expensive than the purely clinical approach and on average even less expensive than in base analysis. This result may be due to the fact that PSA includes all reasonable assumptions, e.g. also a higher influenza prevalence in ILI patients than our low base case assumption of 25.9%. This leads to a higher positive predictive value in favor of rapid influenza testing.

The major portion of the savings is due to the fact that in PSA, the results of which are based on random-sampling and therefore differ from those of the univariate analysis, the proportion of initial unnecessary bed blocking was nearly nine-fold higher (27.9% vs 3.2%) with conventional clinical judgement than with the Sofia^®^. As this mistake can be corrected only after 1–2 days when the result of the PCR is available, the cost difference between the two strategies, with respect to opportunity costs–after exclusion of the costs of Sofia^®^ and of the external PCR–is €113.20 in favor of Sofia^®^.

In PSA, the costs of performing one external PCR are on average €47.44 per tested ILI patient, whereas the costs of rapid testing of the Sofia^®^ are €11.99. However, because the swabs of all 73.21% patients tested negative–65.5% tested correctly negative as well as 7.71% tested falsely negative–have to be re-tested, testing with the Sofia^®^ saves only €0.72 per tested patient compared to conventional clinical judgement.

In PSA, approximately 50% of all 10 000 patients included into the Monte–Carlo simulation, were drawn at random to receive oseltamivir when tested positive by the Sofia^®^ or assumed to have the flu by clinical judgement. Due to the only slightly higher sensitivity of the Sofia^®^, only 2.1% more influenza patients could be properly treated with oseltamivir by using rapid testing than with the conventional strategy (23.6% versus 21.5%), resulting in a lower expenditure with rapid testing of only €1.80 per tested patient.

Finally, lower sick day costs of only €4.17 due to less infected hospital employees per tested patient, at the expenditure of the hospital, is incurred by using Sofia^®^.

## 4. DISCUSSION

Advanced real-time rapid tests such as the Sofia^®^, with specificity exceeding 95%, come close to laboratory PCR tests in their ability to eliminate from consideration for isolation the large percentage of patients presenting in the ER with non-influenza ILI, and that very rapidly. In this they are superior to the conventional clinical approach. However, their sensitivity to detect the influenza virus–75.3 % for the Sofia^®^–is only a few percentage points higher than that of the conventional clinical approach. Thus, the added value of the new tests is low when it comes to deciding whether an ILI patient who is going to be hospitalized due to the severity of his symptoms actually has the flu and should immediately be isolated and treated with oseltamivir. The number of false negative conclusions are nearly identical to the number arrived at by conventional clinical judgement, so that only few more hospital employees will be spared infection due to mistaken influenza status of patients, resulting in a cost saving of only €4.17 per tested patient. The same small difference in sensitivity of 2.1% in favor of the Sofia^®^ enables only few more influenza patients to be promptly treated with oseltamivir and to be discharged 1 day earlier, resulting in a reduction in expenditures of only €1.80 per tested patient. Here, molecular^11^ or isothermal^9^ tests which generally offer a sensitivity of more than 95% have clear advantages over RIDTs and therefore could achieve further cost savings in favor of the hospital.

Also, the significantly lower costs of the rapid test per patient compared to those of an external PCR hardly contribute to reduced expenditures. As in our model 73.2% of our ILI patients are scored negative with the Sofia^®^, adding the costs of the Sofia^®^ itself and those of a subsequently performed external PCR almost completely outweighs the initial cost advantage of the Sofia^®^ test.

The key to the calculated on-average cost saving of €119.89 per patient gained, from the hospital’s perspective, by implementing improved rapid influenza testing lies in shortening the time lag between taking the swabs in the ER and receiving the test result. As the proportion of unnecessary bed blocking at the first day of hospitalization is nearly nine-fold lower with the Sofia^®^ approach, as compared to the conventional clinical approach, lower costs of €113.20, or 94.4% of the total of €119.89 may be achieved through significantly fewer false positive assumptions made regarding the presence of influenza in ILI patients. Each time a patient is wrongly assumed to be suffering from an influenza infection, a hospital’s capacity is reduced, leading to corresponding revenue loss in terms of opportunity costs for the hospital.

Thus, in PSA of our model, performing the Sofia^®^ for influenza suspects leaving the ER for admission to a German hospital ward is consistently less expensive than the conventional symptom-based judgement for which the PCR testing results is only available after 1 or 2 days of delay. Of note, this ranking is not dependent on annual changes of the influenza prevalence in the respective season, at least when–as assumed in our model–influenza prevalence varies between 20% and 42.6%.

Our study has some limitations that must be kept in mind when interpreting our results. As always, the general limitation of a single-center economic model that cannot depict the realities of each single hospital, deserves attention. Thus, although our monocentric model offers advantages for studying the economic consequences of German hospitals that do not utilize rapid influenza testing, it cannot replace empirical statistical data gained by multicentric in-depth investigations. The use of extensive sensitivity analyses is an effort that addresses those limitations. However, to validate our estimates, prospective cost studies, preferably with a multicenter study design, are required.

Furthermore, our calculations refer only to hospitals that must send samples to an external laboratory for influenza testing and wait 1–2 days for the report. Hospitals that have a laboratory department at their disposal that already conducts high quality influenza tests and provides test reports while the patients are waiting in the ER, even during weekend and at night, would probably not benefit from rapid influenza testing.

Another limitation is that our model probably underestimates the true amount of cost savings which may arise from the fact that the test results of the Sofia^®^ as POC test are available in only 3 to 15 minutes.

The Sofia^®^ approach may relieve the ER staff from duties the consequences of which are not often considered. In a recently published economic model incorporating the Alere^®^ Influenza A&B, a molecular test that minimizes the number of ILI patients suspected to have influenza as quickly as does Sofia^®^, Brachman et al.^34^ calculated that by testing 812 influenza suspects in the influenza season 2016/2017 a total of 2733 labor hours could be saved. In particular, routine disinfection of multiple rooms, including the X-ray suite and the examination room, could be prevented by rapidly gaining a (negative) test result. However, as the time portions that the authors added in their calculation are incidental and accrue at different points in time, possible staff services are difficult be operationalized in an economics simulation model.

Another benefit that is probably applicable to other healthcare systems is the option to simultaneously run further viral diagnostics on the same Sofia platform. For example, symptoms of RSV (respiratory syncytial virus) resemble those of influenza in ILI patients, and RSV, like influenza, can spread and lead to pneumonia or exacerbation of chronic obstructive pulmonary disease, especially in older and immune-compromised patients.^35^ According to German guidelines,^5^ isolation of inpatients with pulmonary RSV disease should also be taken into consideration to prevent spread of the virus to co-patients and HCW. Thus, excluding RSV in ER by a POC test, where results can be obtained within the same time frame as for influenza, could further enhance the accuracy of diagnosis and further reduce the number of unnecessary isolation cases and consequent in-hospital costs.^11^

## 5. CONCLUSION

As with molecular or isothermal real-life tests, the utilization of the Sofia^®^ test as an example for an improved rapid POC influenza tests is likely to reduce hospital-related costs in cases of suspected influenza in German emergency rooms. Lowering of expenditures may be achieved, irrespective of varying influenza prevalence in the respective season. In this sense, the results of our analysis may also be directly relevant for other European countries with healthcare systems similar to ours.

## Figures and Tables

**Table 1:** Input for cost–benefit analysis

Variables Category	Variable Name	Distribution*	Value (Base Case)	Relative Change (Range) Univariate	Reference
Prevalence of influenza	Flu_prev	uniform	0.259	0.2–0.426	Calculated from [2]
Additional revenue per day due to NI	cRev_day	triangular	€233.46	±20% (€186.77–€280.15)	Calculated from InEK data [5]
Combined Sofia^®^ specificity	Sofia_spec	uniform	0.953	95% CI (0.915–0.992)	[8]
Opportunity costs due to blocking twin bed	cOpp	triangular	€350.19	±20% (€280.15–€420.23)	Calculated from InEK data [5]
Probability of correctly excluding non-influenza by clinical judgement	Clin_spec	uniform	0.601	95% CI (65.4–76.7)	[22]
Sensitivity of diagnosing influenza if present by clinical judgement	Clin_sens	uniform	0.713	95% CI (0.65–76.7)	[22]
Costs of NI (oseltamivir) per day	cAntivir_day	triangular	€12.37	±20% (€9.89–€14.83)	Adapted from Rote Liste [Red List] 2019
Costs of Sofia^®^	cSofia	triangular	€12	±20% (€9.6–€14.40)	As declared by manufacturer
Combined sensitivity of the Sofia^®^ test	Sofia_sens	uniform	0.753	95% CI (0.592–0.915)	[8]
Secondary cases due to one unknown influenza case	sec_flu	normal	0.202	95% CI (0.154–0.256)	[23]
Costs of productivity loss per day	cPL_day	triangular	€156.99	±20% (€125.59–€188.39)	calculated from Federal Statistical Office data [24]
Number of days of HCW out of work due to influenza	sick_days	normal	7.2 days	SD 8.9 days (5.76–8.64)	[25,26]
Probability of vaccinated HCW	pVacc_HCW	normal	0.401	±20% (0.33–0.49)	[27]
Probability that hospitalization is required	pHosp	uniform	0.219	0.157–0.23	Adapted from [1]
Costs of PCR in external laboratory	cPCR	triangular	€44.88	€30–€65	Nationwide laboratory inquiry
Effectiveness of influenza vaccination	Vacc_eff	uniform	0.15	0.15–0.49	[1,27]
Probability of administering NI	pNI	uniform	1 or 0	0–1	Model assumption

* in probabilistic sensitivity analysis. SD: Standard deviation

**Table 2:** Results of the base-case analysis (with and without NI treatment)

*a) with NI treatment*			
Base-Case Analysis	Comparators	Mean Cost Per Patient (€)	Incremental Cost (€)^*^
ILI patients prior to hospitalisation followed by immediate intake of NI	Sofia^®^ as an add-on	114.33	0
	Conventional approach	166.94	52.16
^*^ Incremental cost denotes the increase in total costs resulting from using the conventional approach alone versus including rapid testing.			
***b) without NI treatment***			
**Base-Case Analysis**	**Comparators**	**Mean Cost Per Patient (€)**	Incremental Cost (€)^*^
ILI patients prior to hospitalisation without intake of NI	Sofia^®^ as an add-on	145.65	0
	Conventional approach	193.93	48.28

**Table 3:** Tornado diagram* (rapid POC influenza testing versus the conventional clinical approach)

Variable Name	Variable Description	Variable Lowest Bound	Variable Highest Bound	Lowest Cost Value (€)	Highest Cost Value (€)	Spread (€)τ	Threshold Valueμ	Risk%¥	Cumulative Risk%
Sofia_spec	Combined specificity of Sofia testing	0.915	0.992	−123.940	16.894	140.83	0.924	0.895	0.895
Sofia_sens	Combined sensitivity of Sofia testing	0.592	0.915	−70.683	−34.645	36.037	-	0.059	0.954
Clin_spec	Probability of correctly excluding non-influenza	0.572	0.630	−62.625	−42.591	20.034	-	0.018	0.972
cOpp	Opportunity costs due to blocking twin bed	280.150	420.230	−62.158	−43.059	19.099	-	0.016	0.988
Clin_sens	Sensitivity of diagnosing influenza if present	0.654	0.767	−58.505	−47.211	11.294	-	0.006	0.994
cPCR_ext	Costs of PCR in external laboratory	30.000	65.000	−57.233	−49.188	8.045	-	0.003	0.997
cSofia	Costs of Sofia test consumables inclusive	9.600	14.400	−55.008	−50.208	4.800	-	0.001	0.998
Flu_prev	Prevalence of influenza	0.200	0.426	−53.833	−49.142	4.690	-	0.001	0.999
pNI	Probability of NI treatment in per cent	0.000	1.000	−52.608	−48.282	4.326	-	0.001	1
sec_flu	Secondary cases due to one unknown influenza case	0.154	0.256	−53.202	−52.081	1.121	-	0	1
cRev_day	Additional revenue per day due to NI	186.770	280.150	−53.092	−52.125	0.967	-	0	1
cPL_day	Costs of productivity loss per day	125.590	188.390	−53.052	−52.164	0.888	-	0	1
sick_days	Number of days of HCW out of work due to influenza	5.760	8.640	−53.052	−52.164	0.888	-	0	1
cAntivir_day	Costs of virustatics per day	9.890	14.830	−52.988	−52.226	0.762	-	0	1
Vacc_eff	Effectiveness of influenza vaccination	0.150	0.490	−52.608	−52.278	0.330	-	0	1
pVacc_HCW	Probability of HCW	0.328	0.492	−52.637	−52.579	0.058	-	0	1
pHosp	Probability that hospitalization is required	0.157	0.230	−52.608	−52.608	0	-	0	1

* One-way sensitivity analyses of all model variables arranged in order, with the variable with the biggest impact at the top and the variable with the smallest impact at the bottom;

¥ Risk%: This is a measure of how much of the total uncertainty is represented by the respective variable. The Risk% values sum to 1.0 across all the variables;

τ Highest cost value minus lowest cost value;

μ Indicates the point at which absolute savings turn to expenditures.

HCW: health care workers.

**Table 4:** Results of the probabilistic sensitivity analysis (Monte Carlo Simulation)

Probabilistic Sensitivity Analysis	Comparators	Mean Cost Per Patient (€)	Standard Deviation (SD)	Incremental Cost (€)*
ILI patients prior to hospitalisation, one half followed by intake of NI	Sofia^®^ as an add-on	56.65	17.28	0
	Conventional approach	176.54	22.48	119.89

* Incremental cost denotes the increase in total costs resulting from using the conventional approach alone versus including rapid testing.
